# Supplementary material for: Pathogenic LRRK2 mutations cause loss of primary cilia and Neurturin in striatal parvalbumin interneurons
Source: Life Sci Alliance. 2024 Nov 13;8(1):e202402922. doi: 10.26508/lsa.202402922 (PMC11561259; doi:10.26508/lsa.202402922)
Supplement: Supplementary file 1 [file LSA-2024-02922_TableS1.docx]

**Supplementary Table 1. Stereological Parameters Employed.**

| Genotype | # section | Section interval | Height of disectors (μm) | Guard zone height (μm) | Counting frame  area  (μm) | Sampling grid area (μm) | Measured section thickness mean (μm) | Coefficient error  (CE) |
| --- | --- | --- | --- | --- | --- | --- | --- | --- |
| Wild type | 10 | 6 | 30 | 3 | 100×100 | 400×400 | 27 | CE_m=1_0.06 ≤ CE ≤ CE_m=0_0.09 |
| R1441C | 10 | 6 | 30 | 3 | 100×100 | 400×400 | 27 | CE_m=1_0.06 ≤ CE ≤ CE_m=0_0.09 |
