# Supplementary material for: Pathogenic LRRK2 mutations cause loss of primary cilia and Neurturin in striatal parvalbumin interneurons
Source: Life Sci Alliance. 2024 Nov 13;8(1):e202402922. doi: 10.26508/lsa.202402922 (PMC11561259; doi:10.26508/lsa.202402922)
Supplement: Supplementary file 2 [file LSA-2024-02922_TableS2.docx]

**Supplementary Table 2. Area analyzed for each Lenticular nucleus tissue sample**

| Brain ID | Tiles analyzed | Area analyzed (mm^2^) | PV neurons |
| --- | --- | --- | --- |
| 01-31 (control) | 493 | 41.38 | 264 |
| 01-46 (control) | 508 | 44.53 | 270 |
| 03-63 (control) | 596 | 52.86 | 218 |
| 18-42 (control) | 415 | 36.72 | 188 |
| 10-28 (sporadic PD) | 447 | 39.38 | 125 |
| 16-23 (sporadic PD) | 390 | 34.98 | 129 |
| 18-72 (sporadic PD) | 427 | 38.75 | 137 |
| 21-81 (sporadic PD) | 253 | 22.74 | 85 |
| 01-39 (G2019S) | 513 | 44.92 | 137 |
| 10-37 (G2019S) | 228 | 20.36 | 58 |
| 13-60 (G2019S) | 424 | 37.83 | 110 |
