# Supplementary material for: Pathogenic LRRK2 mutations cause loss of primary cilia and Neurturin in striatal parvalbumin interneurons
Source: Life Sci Alliance. 2024 Nov 13;8(1):e202402922. doi: 10.26508/lsa.202402922 (PMC11561259; doi:10.26508/lsa.202402922)
Supplement: Supplementary file 3 [file LSA-2024-02922_TableS3.docx]

**Supplemental Table 3. Key Resources used in this study**

| **Reagent type (species) or resource** | **Designation** | **Source or reference** | **Identifiers** | **Additional information** |
| --- | --- | --- | --- | --- |
| Genetic reagent (Mus musculus) | Constitutive KI Lrrk2tm4.1Arte | Taconic | #13940, RRID:IMSR_TAC:13940 | C57BL/6; G2019S KI |
| Genetic reagent (Mus musculus) | Constitutive KI Lrrk2tm1.1Shn/J | Jackson Laboratory | #009346, RRID:IMSR_JAX:009346 | C57BL/6; R1441C KI |
| Antibody | anti-Parvalbumin  (guinea pig polyclonal) | Synaptic system | #195004  (RRID:AB_2156476) | (1:1000 for mice brains)  (1:400 for human brains) |
|  |  |  |  |  |
| Antibody | anti-Somatostatin 28  (guinea pig polyclonal) | Synaptic system | #366004  (RRID:AB_2620126) | (1:500) |
|  |  |  |  |  |
| Antibody | anti-cKit (D13A2) XP  (rabbit monoclonal) | Cell signaling | #3074  (RRID:AB_1147633) | (1:1000) |
|  |  |  |  |  |
| Antibody | anti-D1DR  (rat monoclonal) | Sigma-Aldrich | D2944  (RRID:AB_1840787) | (1:500) |
|  |  |  |  |  |
| Antibody | anti-Adenylate cyclase III (rabbit polyclonal) | EnCOR | RPCA-ACIII  (RRID:AB_2572219) | (1:10000) |
|  |  |  |  |  |
| Antibody | anti-Adenylate cyclase III  (mouse monoclonal) | Santa Cruz | SC-518057  (RRID:AB_3073967) | (1:100) |
|  |  |  |  |  |
| Antibody | anti-DRPP32  (rabbit monoclonal) | Cell Signaling Technology | #2306S  (RRID:AB_823479) | (1:400) |
|  |  |  |  |  |
| Antibody | H+L Donkey anti-guinea pig Alexa 647 | Jackson ImmunoResearch | #706-605-148  (RRID:AB_2340476) | (1:2000) |
|  |  |  |  |  |
| Antibody | H+L Donkey anti-guinea pig CF^TM^ 488A | Sigma-Aldrich | SAB4600033  (RRID:AB_2890881) | (1:2000) |
|  |  |  |  |  |
| Antibody | H+L Donkey anti-Rabbit Alexa 568 | Life Technologies | A10042  (RRID:AB_2534017) | (1:2000) |
|  |  |  |  |  |
| Antibody | H+L Donkey anti-Rat Alexa 647 | Jackson ImmunoResearch | #712-605-153  (RRID:AB_2340694) | (1:2000) |
|  |  |  |  |  |
| Antibody | H+L Donkey anti-mouse Alexa 647 | Life Technologies | A31571  (RRID:AB_162542) | (1:2000) |
|  |  |  |  |  |
| Commercial assay or kit | RNAscopeMultiplex  Fluorescent Reagent Kit v2 | Advanced Cell Diagnostics | #323100 |  |
|  |  |  |  |  |
| Commercial assay or kit | RNAscope Probe-Mm-Lrrk2 | Advanced Cell Diagnostics | #421551 | (1:20) |
| Commercial assay or kit | RNAscope Probe-Mm-Gli1 | Advanced Cell Diagnostics | #311001 |  |
| Commercial assay or kit | RNAscope Probe- Mm-Ptch1-C2 | Advanced Cell Diagnostics | #402811-C2 | (1:5) |
| Commercial assay or kit | RNAscope Probe- Mm-Nrtn-C2 | Advanced Cell Diagnostics | #441501-C2 | (1:3) |
| Commercial assay or kit | OPAL 690 REAGENT PACK | Akoya Biosciences | FP1497001KT |  |
| Software, Algorithm | FIJI | PMID:29187165 | RRID:SCR_002285 |  |
| Software, Algorithm | Prism | Prism 9 version 9.3.1 | RRID:SCR_002798 |  |
| Software, Algorithm | ZEN | ZEISS ZEN  Microscopy  Software | RRID:SCR_013672  <https://www.zeiss.com/microscopy/en/products/software/zeiss-zen.html> |  |
|  |  |  |  |  |
| Software, Algorithm | Stereo Investigator | MBF Bioscience | RRID:SCR_002526  <https://www.mbfbioscience.com/products/stereo-investigator> | Version 2024.1.2 |
|  |  |  |  |  |
